# Progress of Nonmetallic Electrocatalysts for Oxygen Reduction Reactions

**DOI:** 10.3390/nano13131945

**Published:** 2023-06-26

**Authors:** Zhongmei Che, Yanan Yuan, Jianxin Qin, Peixuan Li, Yulei Chen, Yue Wu, Meng Ding, Fei Zhang, Min Cui, Yingshu Guo, Shuai Wang

**Affiliations:** 1 Shandong Provincial Key Laboratory of Molecular Engineering, School of Chemistry and Chemical Engineering, Qilu University of Technology, Shandong Academy of Sciences, 3501, Daxue Road, Changqing District, Jinan 250353, Chinacuimin@qlu.edu.cn (M.C.); 2 Qingdao Haiwang Paper Co., Ltd., 1218, Haiwang Road, Huangdao District, Qingdao 266431, China

**Keywords:** nonmetallic electrocatalysts, oxygen reduction reaction, carbon materials, doping, prospect

## Abstract

As a key role in hindering the large-scale application of fuel cells, oxygen reduction reaction has always been a hot issue and nodus. Aiming to explore state-of-art electrocatalysts, this paper reviews the latest development of nonmetallic catalysts in oxygen reduction reactions, including single atoms doped with carbon materials such as N, B, P or S and multi-doped carbon materials. Afterward, the remaining challenges and research directions of carbon-based nonmetallic catalysts are prospected.

## 1. Introduction

Fuel cells and metal-air batteries are clean, efficient and safe batteries that convert chemical energy directly into electric energy, and they have the advantages of a high energy density, safety and environmental protection compared with traditional energy sources [1,2]. Currently, Pt-based cathode catalysts are widely used as the main catalyst of precious metals; however, high costs and limited reserves become major obstacles to the commercialization of fuel cells [3,4,5]. In addition, the fuel usually contains an amount of CO gas, easily making a Pt-based catalyst susceptible to poisoning [6,7,8]. Direct methanol fuel cells are a type of fuel cells that use methanol as the anode active substance directly. In these cells, methanol can penetrate the cathode through an ion exchange membrane, leading to catalyst poisoning and deactivation. Hence, studying the methanol tolerance of catalysts is of significance in practical applications. Therefore, improving the utilization rate of platinum, reducing its consumption and developing new non-platinum catalysts with low prices have become the main research directions of electrocatalysts for low-temperature fuel cells in recent years [9,10,11].

The development of low-cost, highly reactive non-precious metal catalysts can reduce the cost of fuel cells and promote large-scale commercial applications [7,12,13,14,15]. The surface chemical properties of one-dimensional titanates give them broad prospects in catalysis and energy storage [16]. Recent studies show that nonmetallic catalysts have a relatively high ORR catalytic activity and stability, and they are expected to replace Pt-based catalysts [6,17,18,19,20,21]. For ORR catalysts, the following basic conditions must be met: (1) good electrical conductivity; (2) high specific surface area; (3) abundant active sites [22]. Therefore, heteroatomic-doped nano-carbon materials are the most studied among non-metallic catalysts, including single-atom (such as N, S, P and B) doped carbon materials and multi-doped (such as N/S, N/B, N/P and N/P/S) carbon materials [6,12,23,24,25,26,27]. From the above two aspects, this paper mainly summarizes the preliminary research progress of nonmetallic catalysts in oxygen reduction reactions, aiming at reducing the cost of cathode catalysts, improving the activity and stability of catalysts and prospecting the future research direction of non-platinum-based oxygen reduction catalysts.

## 2. Carbon Materials

### 2.1. Carbon Nanotubes

Carbon nanotubes are one-dimensional materials with a special structure (nanoscale radial dimensions, micrometer-scale axial dimensions and sealed ends at both ends of the tube). They are well known for their excellent mechanical strength, stability and conductivity and large specific surface areas, making them highly favored in the field of electrochemistry as electrode materials [28]. The tube ends and defects of carbon nanotubes have a certain catalytic activity and can directly catalyze oxygen reduction. The surface modification of carbon nanotubes can significantly improve their catalytic performance, and the current approach for catalyzing oxygen reduction mainly involves functionalizing their surface groups and doping them with oxygen or nitrogen atoms to promote the oxygen reduction reaction [29].

### 2.2. Graphene

Graphene is a novel carbon material composed of a single atomic layer of sp^2^ hybridized carbon atoms. Due to its large specific surface area, excellent conductivity, high electron transfer efficiency and good chemical stability, it can be used as a promising catalyst support material for the cathodic oxygen reduction reaction in fuel cells [30,31,32]. Graphene doping with heteroatoms (such as N, P, S, etc.) can not only change the charge distribution and electronic properties in the graphene structure but also create new active centers on its surface, thereby enhancing its electrocatalytic activity [33,34]. In addition, the doped atoms are covalently bound to the carbon atoms, and the effect of graphene doping will not fade away, even in a long-term battery environment, ensuring the stability and practical value of doping as a cathodic ORR catalyst.

### 2.3. Graphdiyne

Graphdiyne is a two-dimensional planar carbon material formed by connecting benzene rings through 1,3-diynyl bonds. Its sp^2^ and sp hybridized carbon structures give it high π-conjugation, a regularly ordered porous structure and tunable electronic properties, making it potentially useful in the field of energy storage. The unique sp-C in GDY provides specific sites for designing chemical reactions, enabling controlled doping. The presence of diynyl bonds in GDY creates positively charged carbon atoms, promoting the adsorption and activation of oxygen and facilitating its decomposition. The presence of sp-hybridized carbon atoms in GDY allows for the further regulation of the charge distribution through the doping of heteroatoms (such as N, S, B, P, etc.), generating new hybrid forms and improving electrocatalytic activity.

## 3. Single-Atom Doped Carbon Materials

### 3.1. Nitrogen Doping

**Figure 1:**
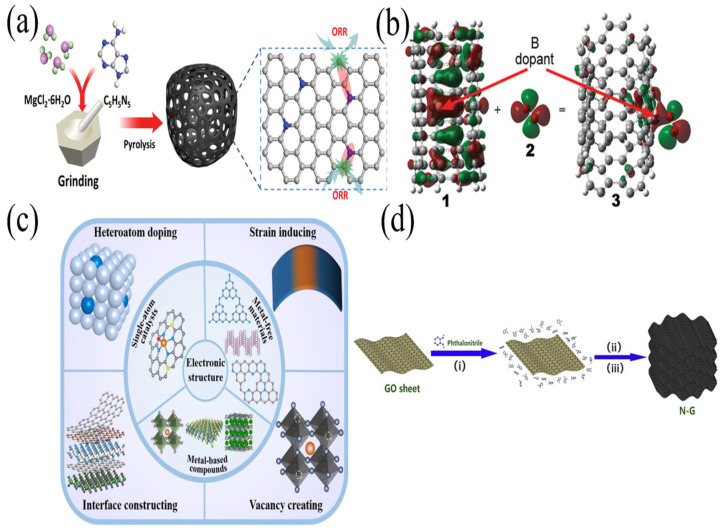
(**a**) Schematic illustration for the synthesis of NDPC [9]. (**b**) Schematic of the molecular orbits involved in the O_2_ adsorption on B-doped CNT. 1, 2 and 3 are the spin-down HOMO-1 of B-doped CNT (5,5), LUMO of the triplet O_2_ and spin-down HOMO-2 of O_2_ adsorbed on the B-doped CNT (5,5), respectively [35]. (**c**) Schematic illustration showing the electronic structure regulation strategies for different noble metal-free electrocatalysts [36]. (**d**) Schematic illustration for the fabrication process of nitrogen-doped grapheme aerogels. (i) Mixing GO with o-phthalonitrile; (ii) Solvothermal treatment of the resulting mixture; (iii) Thermal treatment of the composites from step (ii).

**Figure 2:**
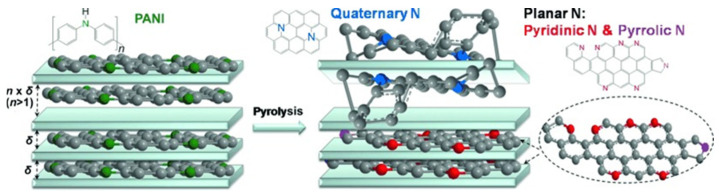
Schematic representation showing the selectivity inside and outside of MMT during NG synthesis [40].

The process of nitrogen (N) doping carbon materials is relatively simple and easy to achieve, and the ORR catalytic activity and stability of N-doped carbon materials are high. Therefore, as depicted in Figure 1, among all kinds of heteroatomic-doped carbon materials, N-doped carbon materials are the most studied [9,35,36,37]. The doping structure of nitrogen atoms plays a leading role in the performance of the catalyst. N-doped carbon materials have five bonding forms, which are pyridine nitrogen, pyrrole nitrogen, graphite nitrogen, nitrile nitrogen and nitrogen oxide [38]. It is not clear which N-doped carbon material has the best activity [39]. As shown in Figure 2, Ding et al. believe that pyridine nitrogen and pyrrole nitrogen have two-dimensional planar structures, while graphite nitrogen shows a three-dimensional uneven structure [40]. When the N doping concentration is low, it is easy to form a three-dimensional uneven structure of graphite nitrogen, destroy the original conjugated large π bond of graphene, make the electrical conductivity of N-doped graphene materials deteriorate and reduce the catalytic activity of ORR.

When the N doping concentration is high, it is easy to form pyridine nitrogen and pyrrole nitrogen with a two-dimensional planar structure; thus, the plane-conjugated large π bond structure can be preserved. The electrical conductivity of N-doped graphene materials is higher, so the catalytic activity of ORR is better. The direction of preparing high-performance N-doped carbon materials is to form pyridine nitrogen and pyrrole nitrogen with a two-dimensional planar structure and reduce or inhibit graphite nitrogen with a three-dimensional uneven structure [41]. The N content and specific surface area of doped carbon materials have an important effect on the catalytic performance. The template method can increase the specific surface area of the catalyst.

**Figure 3:**
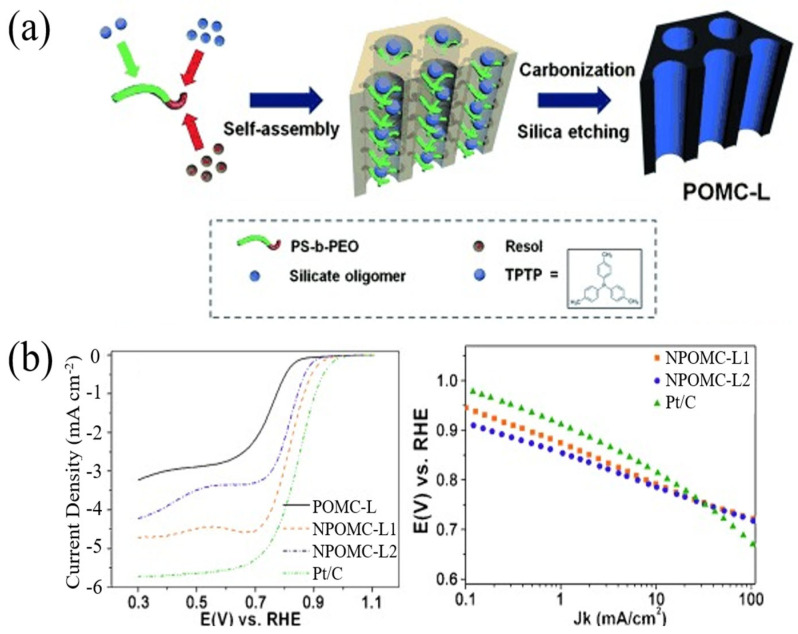
(**a**) Representation of the synthesis of POMC-L. (**b**) LSV polarization curves and corresponding Tafel plots [30].

As shown in Figure 3a, Huang et al. used melamine fiber as a template to prepare an N-doped carbon nanoribbon (NCNR) catalyst [42]. Through the synergistic effect of the N atom and carbon nanoribbon structure, the NCNR catalyst shows high ORR activity due to its high nitrogen content and large specific surface area. Compared with the commercial 20% Pt/C catalyst, the NCNR also has better long-term stability and tolerance to methanol (Figure 3b).

**Figure 4:**
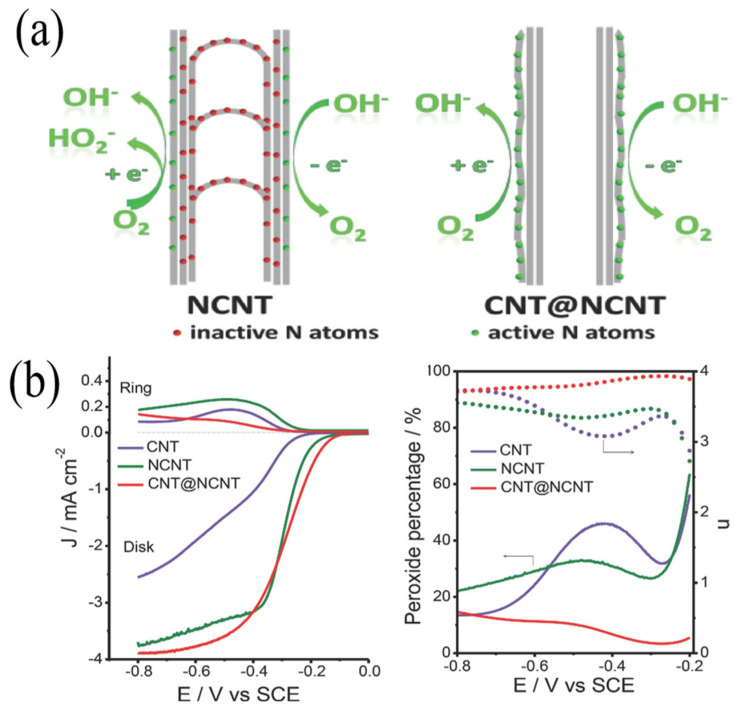
(**a**) Scheme for the full exposure of ‘active sites’ on the surface: NCNTs with the bulk doping of nitrogen atoms and CNT@NCNT coaxial nanocables with surface-enriched nitrogen for OER and ORR. (**b**) Rotating ring disk voltammograms recorded for CNT, NCNT and CNT@NCNT electrodes in an O_2_-saturated 0.1 M KOH solution at a scan rate of 5.0 mV s^−1^, and the percentage of peroxide (solid line) and the electron transfer number (n, dotted line) of the CNT, NCNT and CNT@NCNT catalysts at various potentials, based on the corresponding RRDE data [48].

Carbon nanotubes (CNTS) have many advantages, such as an intrinsic sp hybrid structure, excellent electrical conductivity, high specific surface area and good chemical stability, which have attracted wide attention [43]. Therefore, the doping of heterogeneous elements in CNTs can optimize the surface electronic structure and surface charge distribution of the material, enhance the adsorption of O_2_, and also introduce more defects (edges, vacancy, etc.) to promote ORR catalysis [44]. Nitrogen-containing precursors come from a wide range of sources and have a controllable structural morphology, so N-doped carbon nanotubes (N-CNTs) have attracted widespread attention. Dai et al. prepared electron-rich N-doped sp^2^ hybrid vertical carbon nanotube arrays (VA-NCNTs), and the ORR activity was close to that of P/C under alkaline conditions [45]. As N atoms possess a higher electronegativity than C atoms (the N electronegativity is 3.04; the C electronegativity is 2.55), a theoretical calculation shows that N doping makes adjacent c atoms positively charged, which is conducive to the adsorption of O_2_ for promoting ORR activity [46]. The form and content of N bonding have a crucial influence on the catalytic performance. Generally speaking, in N-doped carbon materials, N has three bonding structures, which are pyridine nitrogen (398.6 eV), pyrrole nitrogen (400.6 eV) and graphite nitrogen (401.6 eV). As shown in Figure 4a, Lee et al. prepared a series of N-doped vertical carbon nanotubes and found that their ORR activity was positively correlated with pyridinium nitrogen [47]. The amount of doping was not the only factor affecting N-CNTs, and the accurate doping of N atoms to the target position was the primary condition for improving activity (Figure 4b). Wei et al. prepared a composite catalyst with CNT as the core and N-CNTs as a shell by the CVD method (CNT@N-CNT) [48]. The N element was mainly distributed in the outer shell, and this N-rich shell was easier to fully contact with O_2_, which greatly improved the utilization rate of the active site and showed similar ORR activity to that of Pt/C. In addition, an N-CNTs aerogel was prepared by the method combined with pyrolysis, which showed good catalytic activity and stability due to its high specific surface area (869 m^2^/g), its high electrical conductivity (10.9 S/m) and the porous network structure of the aerogel itself. This work provides a new idea for the preparation of N-CNTs with a high specific surface area. Lee and Zheng, respectively, studied the oxygen reduction catalytic active sites and catalytic mechanisms of nitrogen-doped carbon nanotubes [49,50]. Xiong et al. synthesized nitrogen-doped carbon nanotubes (NCNT) by pyrolysis [51]. The catalytic performance of NCNT was better than that of 20% Pt/C, and the pyrolysis temperature had a great effect on the performance of NCNT. The catalyst obtained at a pyrolysis temperature of more than 900 °C has the best performance, with good catalytic activity and stability.

**Figure 5:**
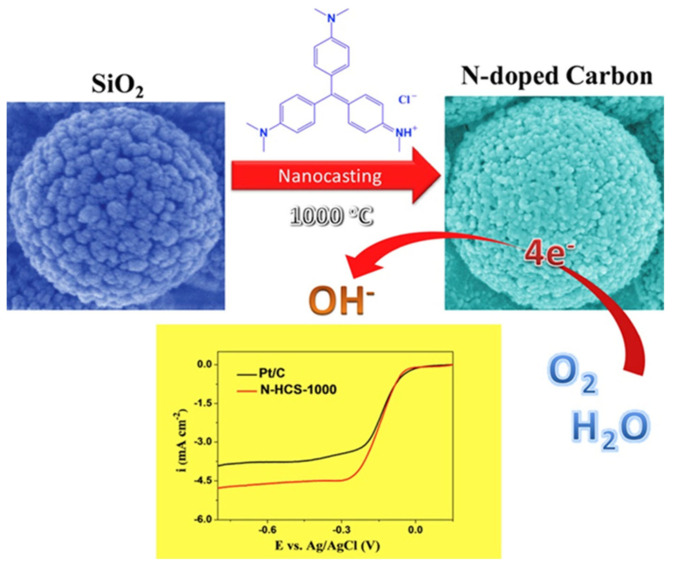
Scheme for N-HCs and LSV curves for ORR performance [52].

Furthermore, as shown in Figure 5, Liu et al. prepared N-doped layered carbon microspheres (N-HCs) by nano-etching using layered mesoporous silica spheres as hard templates and methyl violet as C and N sources [52]. The specific surface area of N-HC_S_ is up to 1413 m^2^/g, and it has a high ORR catalytic activity and current density in an alkaline medium. The initial potential is close to that of commercial Pt/C catalysts.

Meanwhile, the stability and methanol permeability resistance of N-HCs are better than those of Pt/C catalysts. The high ORR catalytic activity of N-HC_S_ is due to the high electrical conductivity and layered porous structure of N-doped carbon-based materials [9,40]. It is easy to dope in an environment with sufficient N sources; the catalytic activity, the stability of N-doped carbon catalysts and the ORR performance are better than those of N-doped carbon graphitize [53]. The ORR catalytic activity and stability of N-doped amorphous carbon catalysts are better than those of Fe-N-C catalysts, indicating that the nitrogen content and the content of pyridine nitrogen/graphite nitrogen also affect the catalytic activity of ORR.

**Figure 6:**
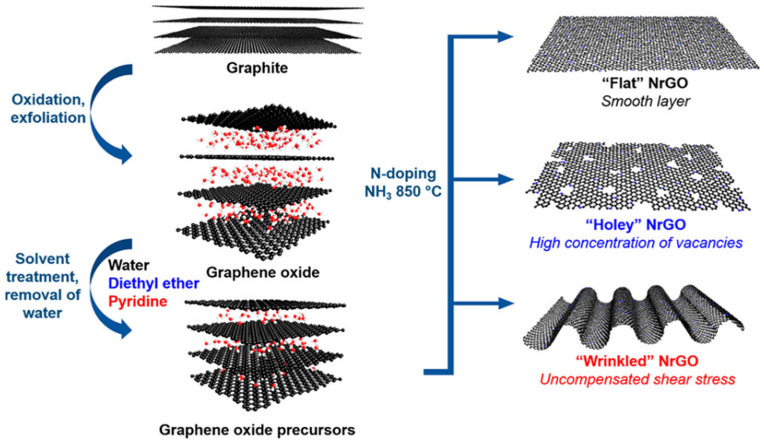
Schematic of the synthesis process. Graphite is (i) exfoliated and oxidized into graphene oxide, (ii) treated with different solvents and dried before (iii) nitrogen doping in a reactive ammonia atmosphere at a high temperature (850 °C). The resulting morphology of the final catalyst is dependent on the solvent used to treat the graphene oxide prior to nitrogen doping [56].

**Figure 7:**
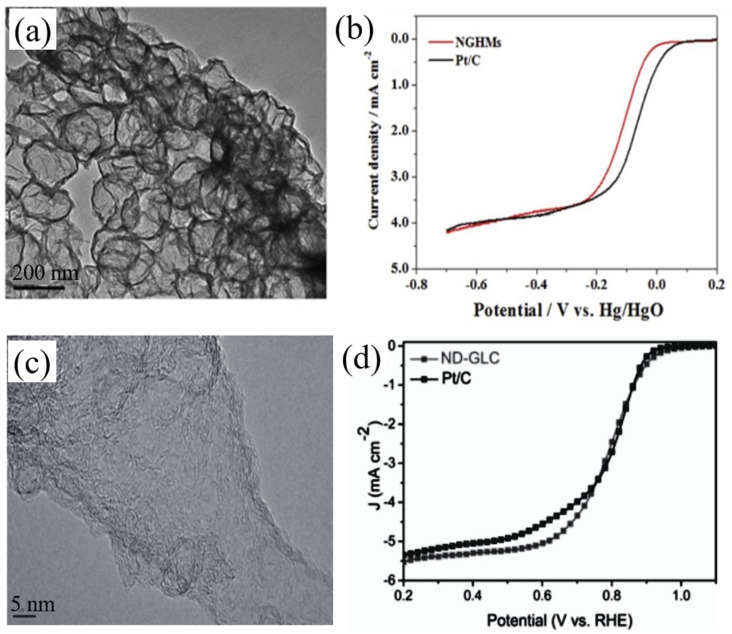
(**a**) TEM images of NGHMs. (**b**) LSV curves of Pt/C (black line) and NGHMs (red line) electrodes in oxygen-saturated 0.1 M KOH solution at a potential scanning rate of 10 mV s^−1^ at 1600 rpm [57]. (**c**) TEM images of ND-GLC. (**d**) LSV curves of Pt/C and ND-GLC [58].

Nitrogen-doped graphene has attracted the investment of many scientific researchers [18,25,54,55]. As shown in Figure 6, Dumont et al. synthesized GO using the modified Hummers method. GO was treated with various solvents, and nitrogen doping was carried out in an ammonia environment with 850 solvents. The morphology of the electrocatalyst changed with different solvents, and the selectivity of ORR was also different. The catalysts should be individually customized [56]. Yan et al. used the Hummers method to produce nitrogen-doped graphene hollow microspheres (NGHMs) by the pyrolysis of a mixture of melamine and polystyrene microspheres coated with graphene oxide (GO) nanosheets in a nitrogen environment (NGHMs), and the N mass fraction of NGHMs reached 7.13% [57]. The TEM results (Figure 7a) clearly showed the hollow spherical structure of NGHMs, compared with the graphene hollow microspheres (GHMS), benefiting the high ORR catalytic activity in the alkaline solution, and the current density was slightly lower than that of commercial 40% Pt/C (Figure 7b). Further kinetic studies showed that the reaction was a four-electron process with a current density, thus demonstrating that the N content and hollow microsphere structure play a major role in the catalytic activity of NGHMs. Liu et al. synthesized nitrogen-doped defective graphene carbon nanomaterials (ND-GLC) catalysts using cellulose as a precursor, combined with high-temperature pyrolysis and ammonia gas treatment [58]. As shown in Figure 7c, SEM and TEM results showed that the structure of ND-GLC catalysts was similar to graphene’s thin-layer carbon nanostructures, and there was a large number of defect sites. A linear sweep voltammetry (LSV) test showed that the catalyst had a better electrocatalytic performance than commercial Pt/C (Figure 7d).

According to the reported research, for the ORR catalytic property with the chemical compositions of N-doped carbon samples, the catalytic activity order of different N species is pyridinic-N > pyrrolic-N > graphitic-N > oxidized-N > C (carbon) [59,60]. Although N-doped carbon materials still face some problems such as unclear catalytic mechanisms and a lower catalytic performance than that of commercial Pt/C catalysts in an acidic medium, they are still one of the non-metallic catalysts with potential development for fuel cells [38,48,61,62,63,64].

### 3.2. Boron Doping

**Figure 8:**
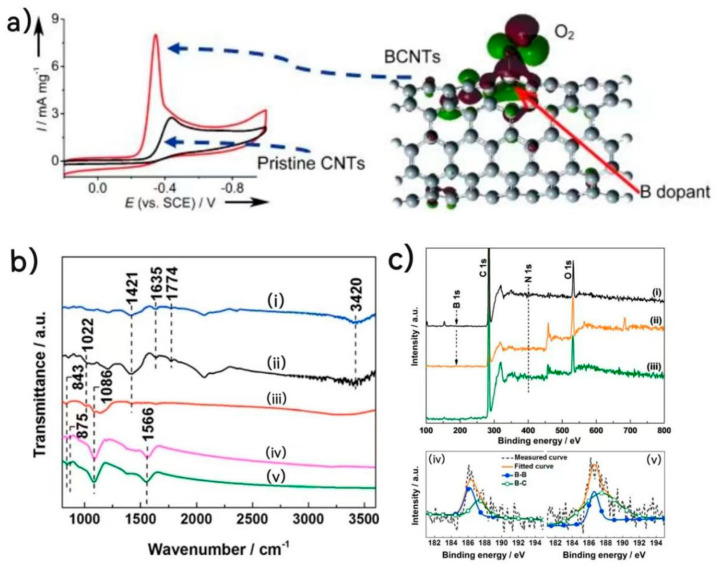
(**a**) CV curves (scan rate 50 mV s^−1^) and the enhanced O_2_ chemisorption and effective utilization of π electrons in the conjugated carbon from the boron doping [67]. (**b**) FT-IR spectra of the deposits for (i) un-annealing and annealing for (ii) 1, (iii) 2, (iv) 3 and (v) 4 h at 1300 °C. (**c**) Survey scanning XPS spectra of the deposits annealed for (i) 1, (ii) 2 and (iii) 3 h, respectively. High-resolution XPS spectra for the B 1 s peak of the deposits annealed for (iv) 1 and (v) 2 h, respectively [68].

Since the boron atom is less electronegative than the C atom, the addition of boron will carry a partial positive charge, while the surrounding C part carries a negative charge [65]. Therefore, the electron-deficient properties of boron can be used to modify pure carbon materials and be used in non-metallic oxygen reduction catalysts. The 2 p_z_ vacant orbital of the B element can conjugate with the delocalized π orbital of carbon to activate the delocalized π electron, strengthen the sp^2^ hybrid structure of graphite and improve the ORR catalytic activity of B-doped carbon materials [66]. As shown in Figure 8a, Yang et al. prepared BCNTs catalysts with B mass fractions of 0.86%, 1.33% and 2.24% by chemical vapor deposition [67]. Under alkaline conditions, the ORR catalytic activity of BCNTs increased with the increase in the B content. The improvement of the ORR catalytic performance was due to the conjugation of the 2 p_z_ orbital of B with the orbital of carbon, which was catalyzed by a two-electron process, similar to N-doped carbon nanotubes 

Compared to those of NCNTs catalysts, the stability and CO toxicity resistance of BCNTs catalysts were also good. However, the ORR catalytic activity of BCNTs was lower than that of commercial Pt/C catalysts and even lower than that of NCNTs catalysts under acidic conditions. Suo et al. made boron-doped carbon catalysts by chemical vapor deposition and high-temperature annealing [68]. As shown in Figure 8b,c, FTIR and XPS showed that, after annealing for 2 h, the boron content was directly proportional to the annealing time, and too long of an annealing time would lead to a decrease in ORR activity. Cyclic voltammetry and rotating disk electrode measurement showed that the catalyst had a better catalytic activity and methanol resistance than Pt/C but a lack of stability. Some scholars have also carried out theoretical simulation studies on boron-doped graphene-related catalysts [69]. Ashraf et al. predicted that boron-doped nanotubes (B-CNT) have a higher ORR performance and efficiency than metal catalysts through DFT calculation [70]. Hu et al. prepared boron-doped carbon nanotubes by chemical vapor deposition [67]. With the increase in boron content, the catalytic activity of oxygen reduction was also increased, revealing the importance of boron. According to theoretical calculations, the positive charge of the boron element is less electronegative than that of carbon and more conducive to the adsorption of oxygen on its surface; thus, the active site of boron can be used as an electron donor in the reduction process to improve the catalytic activity. Subsequently, boron-doped diamonds, carbon nanotubes, graphene sheets and other effective non-metallic oxygen reduction catalysts have been developed [71,72].

### 3.3. Phosphorus Doping

**Figure 9:**
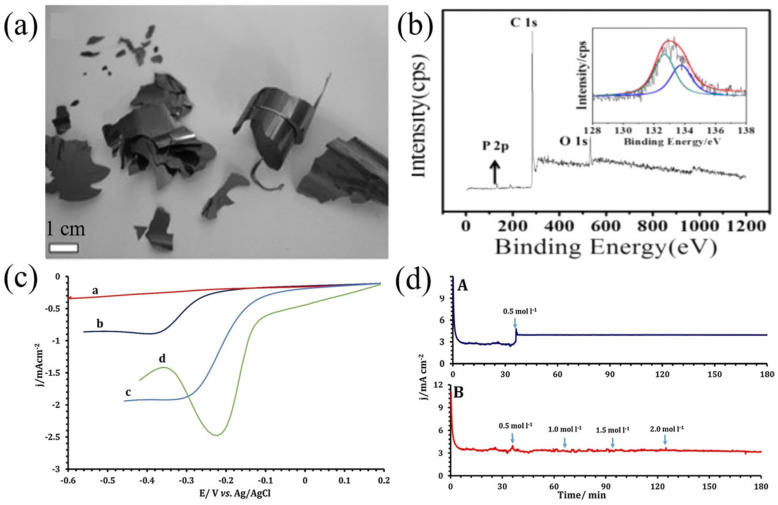
(**a**) Digital photograph of the P-doped graphite layers collected from the quartz tube [73]. (**b**) XPS survey scan and P 2p spectrum (inset) of POMC-3 [74]. (**c**) LSV curves of the ORR at a scan rate of 10 mV s^−1^ at several electrodes in O_2_-saturated 0.1 M KOH solution (a) unmodified GCE, (b) rGO modified GCE, (c) GO-PPh_2_ modified GCE, (d) Pt-rGO modified GCE [75]. (**d**) Chronoamperometry carried out on (A) Pt-rGO, (B) GO-PPh_2_: modified GCE in O_2_-saturated 0.1 M KOH solution at 2500 rpm and at a potential of −0.35 V vs. Ag/AgCl, with different concentrations of methanol added to the KOH solution to estimate the crossover effects [75].

Theoretical calculations have confirmed that the involvement of phosphorus can effectively promote oxygen reduction catalysts. Peng et al. prepared phosphorus-doped graphite flake materials, which showed high electrocatalytic activity, stability and methanol resistance in alkaline dielectrics [73]. As shown in Figure 9a, phosphorus-doped multi-walled carbon nanotubes were obtained by adding ferrocene to the original system, and the diameter and size of the nanotubes could be controlled by the pyrolysis temperature. The catalytic activity of carbon nanotubes containing a small amount of phosphorus exceeds that of commercial carbon-loaded platinum under alkaline conditions, but the role of residual metal has yet to be investigated. Moreover, Yang used the SBA-15 template to prepare phosphorus-doped mesoporous carbon materials, ensuring that the catalyst only acts as phosphorus and thus avoids the effect of metal on the oxygen reduction activity [74]. As revealed in Figure 9b, X-ray photoelectron spectroscopy (XPS) confirmed that phosphorus mainly exists in the form of P-O and P-C in the catalyst doped with phosphorus. The doping of P has significantly improved the catalytic activity of pure carbon materials and is more stable and more resistant to toxicity than commercial Pt/C.

The P-doped reduced graphene oxide (P-RGO) catalyst prepared by Liu et al. [73] has better stability and a stronger ability to resist CO poisoning than the commercial Pt/C catalyst. Ensafi et al. treated a graphene oxide (GO) base with potassium hydroxide and synthesized phosphorus-functionalized graphene oxide (Go-PPh_2_) by reacting a hydroxy-functionalized graphene substrate with chlorobenzene phosphate (ClPPh_2_), which has good ORR catalytic activity and methanol (ethanol) resistance, higher selectivity, better durability and better electrochemical stability than the commercial Pt/C catalyst (Figure 9c,d) [75]. Quilez-Bermejo et al. studied the effect of phosphorus doping on ORR by synthesizing polyaniline-containing phosphorus groups and found that the presence of phosphorus can significantly improve the catalytic activity [54]. Puziy et al. studied the preparation, properties and application of phosphorus-containing carbon and found that phosphorus-containing carbon has chemical stability, and acidic species containing phosphorus have more advantages in electrochemical applications and fuel cells [76].

Although the ORR catalytic activity of B- and P-doped carbon catalysts is not as good as that of commercial Pt/C catalysts, they have better stability and anti-CO poisoning ability than Pt/C catalysts, which is of certain value in the research of non-metallic catalysts [77].

### 3.4. Sulfur Doping

**Figure 10:**
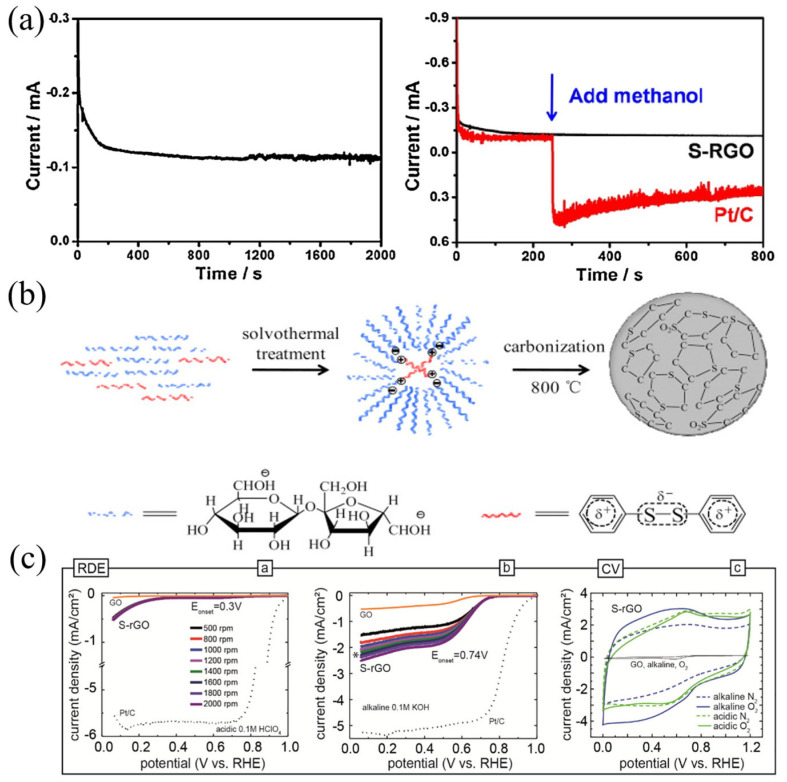
(**a**) The steady-state chronoamperometric response at the polarizing potential of −0.3 V in the O_2_-saturated electrolyte for S-RGO catalysts; methanol crossover tests introducing 1.0 mL of methanol into the electrolyte at 250 s of S-RGO and Pt/C catalysts during the steady-state chronoamperometric response [78]. (**b**) Schematic illustration of the preparation of S-doped carbon spheres [79]. (**c**) LSV in the RDE of S-rGO in (a) acidic and (b) alkaline electrolytes. The star in (b) marks the respective LSV for S-rGO at 1600 rpm for better comparison; (c) shows cyclic voltammograms of S-rGO in N_2_ and O_2_ saturated acidic and alkaline electrolytes, respectively [81].

In addition to studying doping N, B or P, the sulfur atom, which is more electronegative than B and P and similar to C, has also received much attention. Zhai et al. prepared S-doped reduced graphene oxide (S-RGO) nanosheets using dimethyl sulfone and GO as raw materials by a simple method [78]. Compared with the commercial 20% Pt/C catalyst, S-RGO has better corrosion resistance to methanol, stability and CO poisoning resistance (Figure 10a). As shown in Figure 10b, Sun et al. prepared S-doped carbon microspheres with a micropore structure by the in situ doping method, with a specific surface area of more than 503 m^2^/g [79]. The ORR catalytic activity and stability of S-doped carbon microspheres are better than those of undoped carbon microspheres. The reason is that the crystal structure and specific surface area of microspheres are changed by S doping into an appropriate carbon lattice, and the content of S plays a key role in the catalytic activity of the ORR of doped carbon microspheres [80]. Klingele et al. studied the ORR catalytic activity of S-doped carbon materials in acidic and alkaline electrolytes, and Figure 10c shows that, in S-doped reduced graphene oxide (S-RGO), when the mass fraction of S is about 2.2%, the initial potential of oxygen reduction in acidic and alkaline electrolytes is 0.30 V and 0.74 V, respectively [81]. Ma et al. used continuous charge–discharge cycles of graphene–sulfur composites in Li-S batteries to prepare sulfur-doped graphene catalysts, which showed better ORR activity than that of pure graphene and better alcohol tolerance than that of commercial Pt/C [82].

Li et al. prepared sulfur-doped graphene according to the solid phase reaction between graphene oxide and sulfate, and the oxidized sulfur could be transformed into thiophene sulfur with electrochemical activity. 

**Figure 11:**
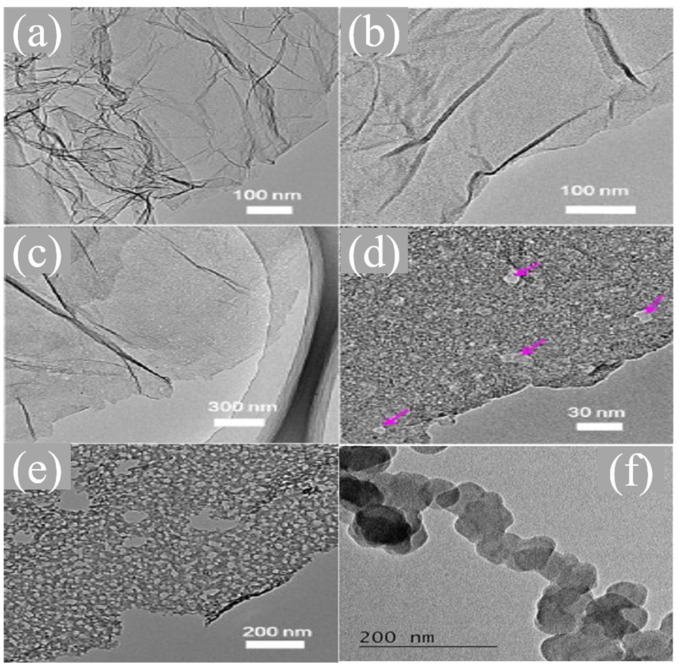
TEM images of (**a**) rGO700, (**b**) S-rGO500, (**c**) S-rGO700, (**d**) the enlarged image of S-rGO700 (pores are marked by arrows) and (**e**) S-rGO1000 [84]. (**f**) HRTEM image of SDC [83].

As shown in Figure 11a, TEM showed that the heating temperature and sulfate dosage would affect the sulfur doping type, doping level and porous structure [83]. Kamaraj et al. reported a kind of sulfur-doped carbon nanoparticle (SDC) with a linked chain structure. As demonstrated in Figure 11b, TEM analysis found that the connected sulfur-doped carbon nanoparticles were a chain-like structure, which could enhance the electrical conductivity and electrolytic activity of the electrode. Cyclic voltammetry showed that the material showed superior ORR stability in acidic media [84]. Recently, Chen et al. synthesized sulfur-doped graphene foams by one-step solvothermal in situ sulfur doping [85]. Zhang explained the catalytic mechanism of sulfur doping by a theoretical calculation and experiment. Sulfur doping enhanced the charge and spin density of carbon atoms around sulfur, which was conducive to oxygen adsorption and the four-electron reduction process [86]. Zhang et al. prepared 3D sulfur-doped graphene with 723-type sulfuric acid-based acid ion-exchange resin with a very high specific surface area and crystallinity [87]. Under alkaline conditions, the catalytic activity can be compared with that of commercial Pt/C and has a higher stability and methanol toxicity resistance.

Although S-doped carbon catalysts have good corrosion resistance, stability and CO poisoning resistance to methanol, the catalytic activity of ORR in acidic and alkaline electrolytes needs to be improved.

## 4. Binary-Doped Carbon Materials

In addition to doping carbon materials with a single element, the binary doping of carbon materials can also be performed using synergies between heteroatoms, such as nitrogen, boron, nitrogen, sulfur, nitrogen and phosphorus double-doping. The electronegativity of B, P, S and N elements is different from that of N elements. The synergistic effect of these elements with nitrogen and the unique electronic structure can be used to improve the current carbon materials and achieve multi-doping. At present, most of the carbon materials doped with heteroatoms, especially nanotubes and graphene, have been prepared by chemical vapor deposition under vacuum, which is complicated and expensive [88].

### 4.1. Nitrogen Sulfur Double-Doped Carbon Materials

**Figure 12:**
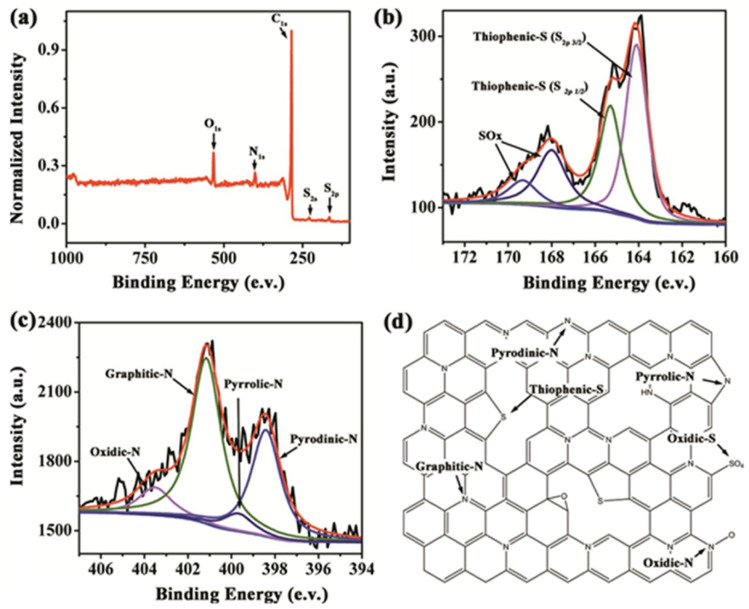
(**a**) XPS survey spectra of the sample ECN-950. (**b**,**c**) High-resolution S 2p and N1s of the sample ECN-950. (**d**) Schematic of heterocyclic N and S structures in the carbon lattice [89].

Jin et al. prepared interlinked nitro sulfur-doped porous carbon nanosheets (SCNs) with glucose/melamine sulfate [89]. As shown in Figure 12, XPS showed that the nitrogen doping concentration was low at high temperatures but reached the highest nitrogen doping level at 950 °C. Rotating electrode measurements showed that SCN-950, with the best performance (synthesized at 950 °C), had similar catalytic activity to that of commercial Pt/C.

Yang et al. designed nitrogen–sulfur co-doped porous carbon plates (NSPCs). According to the results of cyclic voltammetry, although the activity of NSPCs is slightly lower than that of commercial 20% Pt/C, they have a better tolerance to methanol cross-over and show universal pH activity. Qiao et al. synthesized mesoporous thio–nitrogen co-doped graphene carbon materials by a one-step process, which showed good catalytic activity [90]. Liu et al. prepared three-dimensional porous sulfur and nitrogen co-doped carbon foam by using a mixture of cheap mesoporous silicon, sucrose and thiourea through simple high-temperature pyrolysis and HF etching of silicon [91]. These hierarchical three-dimensional porous structures can ensure the electron transfer and reactant transport rate in the oxygen reduction process and improve the stability of the catalyst. The synergistic effect of sulfur and nitrogen makes the catalyst have high catalytic activity. Jin et al. prepared N and S co-doped porous expanded carbon nanosheets (NS-ECNs) with a high specific surface area and large pore volume, which exhibited ideal ORR catalytic activity with a half-wave potential of 0.83 V and initial voltage of 0.97 V [89]. Wang et al. prepared sulfur–nitrogen co-doped ordered mesoporous carbon (SN-OMC) by the silica hard template method [92]. Due to the synergistic effect and ordered mesoporous structure of S and N atoms co-doped in the carbon skeleton, the active sites of the doped carbon materials were increased [93]. The SN-OMC catalyst has the same ORR catalytic activity as the commercial Pt/C catalyst, as well as long-term stability and excellent methanol resistance. Jiang et al. prepared N and S co-doped layered microporous/mesoporous carbon foams (NS-MS) by using pyrolyzed polyaniline-coated spherical sulfur composites [94]. This method is simple, environmentally friendly and free of byproducts. The preparation process does not involve toxic gases such as NH_3_ or H_2_S, nor is an SiO_2_ or MgO template used. NS-MS has good ORR catalytic activity and better methanol resistance and durability than commercial Pt/C, and ORR catalysis is carried out in a four-electron process. By using different sulfur sources and N-CNTs through a hydrothermal method, the S and N-co-doped bamboo carbon nanotubes (SN-CNTs) were prepared; the elements of S and N were evenly dispersed on the surface of the CNTs. In the alkaline electrolyte, the ORR peak potential and peak current of SN-CNTs were better than those of N-doped or undoped CNTs. The ORR performance of SN-CNTs was also significantly improved compared with that of N-CNTs under acidic conditions. This is mainly because the introduction of the S element changes the valence state of N, resulting in the asymmetry of the orbital and charge density and promoting O_2_ adsorption.

### 4.2. Nitrogen–Boron Double-Doped Carbon Materials

**Figure 13:**
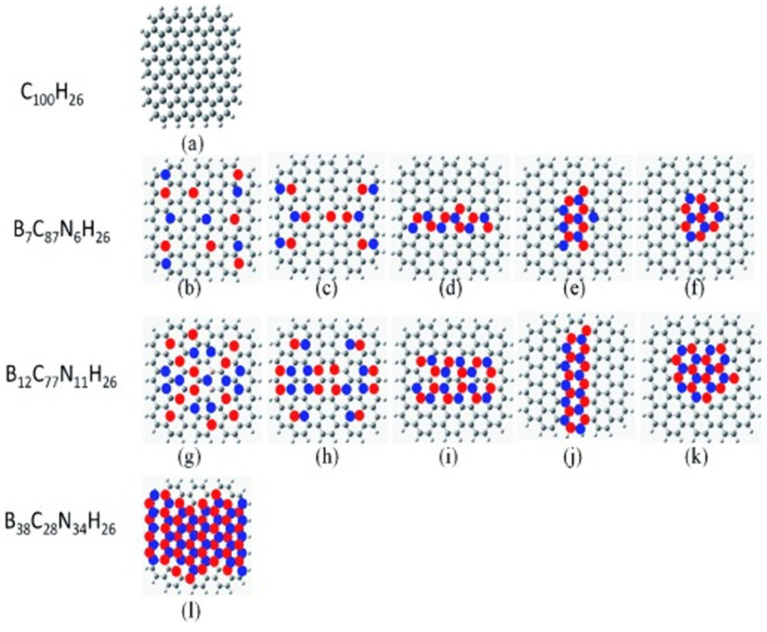
BCN graphene models: (**a**) pure graphene (C100H26), (**b**–**f**) B_7_C_87_N_6_H_26_, (**g**–**k**) B_12_C_77_N_11_H_26_ and (**l**) B_38_C_28_N_34_H_26_. C gray, H white, B pink, N blue [95].

Wang et al. prepared a regulated boron–nitrogen co-doped graphene catalyst with a higher catalytic activity and stability than those of the current commercial Pt/C [95]. As shown in Figure 13a, theoretical calculations revealed that the synergistic effect of boron and nitrogen co-doping can regulate the energy band, spin density and charge density, and the best performance of the catalyst can be obtained by adjusting the doped boron–nitrogen ratio. Jin et al. used cheap urea, boric acid and polyethylene as the precursor system to obtain the folded boron–nitrogen co-doped graphite layered structure, showing a high stable activity, methanol toxicity resistance and four-electron reaction characteristics, confirming that the synergistic effect of two or more elements can better improve the catalytic activity of oxygen reduction catalysts [96]. Zhou et al. prepared B, N co-doped carbon nanotubes (VA-BNCNT) [97]. The N and B-doped binary carbon material system has a broad variety [98]. Xue et al. employed the CVD method to fabricate a nitrogen and boron co-doped graphene foam (BN-GFs), using melamine diborate as a precursor. Electrochemical measurements indicated that the doped graphene foam exhibited significantly higher electrocatalytic activity for the oxygen reduction reaction (ORR) than the undoped counterpart. Additionally, Xu et al. developed a novel N, B-doped graphene aerogel (N, B-GA) via a two-step method. The 3D N, B-GA exhibited excellent catalytic activity for ORR, comparable to that of Pt/C, serving as a non-metal catalyst [2]. The superior catalytic activity is attributed to the synergistic coupling of B and N dopants within the graphene domain.

Compared with single doping, both the initial potential and the limiting diffusion current density of the double-doped product are significantly increased, and the activity is comparable to that of Pt/C, confirming the synergistic effect of double-element doping.

### 4.3. Nitrogen Phosphorus Double-Doped Carbon Materials

**Figure 14:**
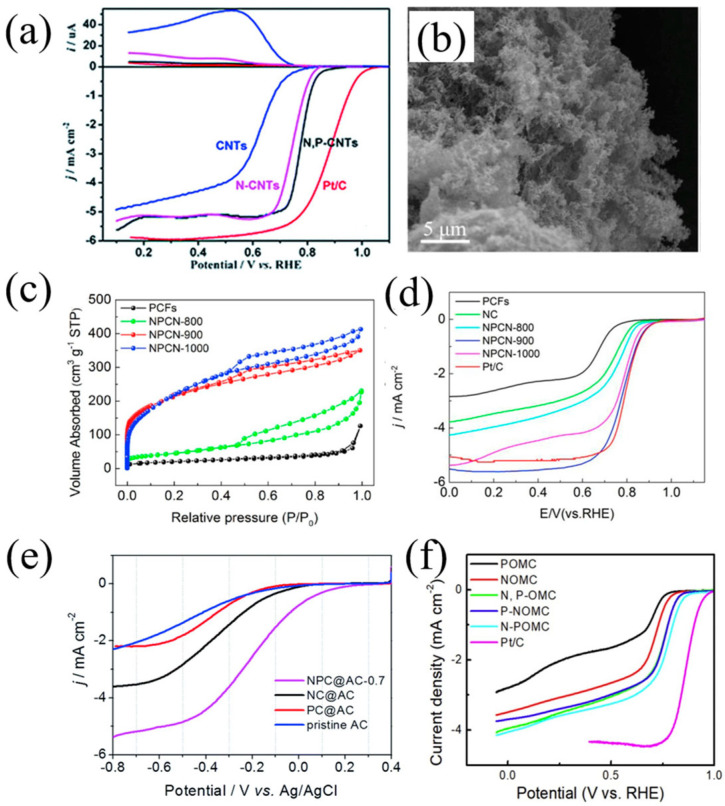
(**a**) The linear sweep curves (down) of the catalysts on RDE for ORR and corresponding ring currents (up) at 1600 rpm in O_2_-saturated 0.1 M KOH at 25 °C with a sweep rate of 5 mV s^−1^ [99]. (**b**) TEM of NPCN-900 [100]. (**c**) N_2_ adsorption/desorption isotherms of PCFs, NPCN-800, NPCN-900 and NPCN-1000. (**d**) LSV curves for the above catalysts and Pt/C in O_2_-saturated 0.1 M KOH with a rotation speed of 1600 rpm [100]. (**e**) LSV curves of NPC@AC-0.7, NC@AC, PC@AC and pristine AC catalysts loaded on RDE at a speed of 1600 rpm in O_2_-saturated neutral 50 mM PBS [101]. (**f**) LSV curves of the as-prepared samples, compared with the commercial Pt/C in oxygen-saturated 0.1 M KOH solution at a scan speed of 10 mV s^−1^ and a rotation speed of 1600 rpm [102].

Zhu et al. developed N-P co-doped carbon nanotubes with large internal channels through a simple one-pot method, allowing oxygen molecules to contact a large number of active sites on the inner wall, thus improving catalytic activity [99]. As shown in Figure 14a, the synergistic effect of nitrogen and phosphorus made the catalyst extremely stable and highly resistant to methanol toxicity. Jiang et al. prepared a N and P co-admixture heterogeneous 3D porous network carbon (NPCN) catalyst [100]. As shown in Figure 14b,c, NPCN shows a unique 3D porous network structure with a specific surface area up to 743 m^2^/ g, and abundant edge defects increase the active site of the material. As depicted in Figure 14d, the initial ORR potentials of NPCN catalysts in alkaline and acidic media are 0.92 V and 0.74 V, respectively, which are close to the 0.93 V and 0.80 V of Pt/C catalysts. NPCN catalysts show better durability and methanol resistance over a wide pH range than commercial Pt/C catalysts. Zhao et al. prepared mesoporous carbon (OMC) with a soluble phenolic resin solution and SBA-15 by the nano casting method (N-POMC) [101]. Electrochemical test results showed that N-POMC exhibited better activity to ORR in alkaline media (Figure 14e), which was attributed to the fact that the first doped P would be replaced by the later doped N by increasing the content of graphite nitrogen. Lv et al. coated polyaniline doped with phytic acid (PA) through an in situ oxidative polymerization reaction and then carried out high-temperature pyrolysis [102]. N and P co-doped carbon was mixed into activated carbon (AC) (represented by NPC@AC). As shown in Figure 14f, compared with single-carbon-doped NC@AC, single-phosphorus-doped PC@AC and pure AC, NPC@AC exhibited higher ORR electrocatalytic activity in a neutral solution.

### 4.4. Multi-Doped Carbon Materials

Although the synergistic effect between heteroatoms can improve the performance of ORR, and the performance is better than that of single-heteroatom-doped non-metallic catalysts, with the development of non-metallic catalysts, multi-doped carbon materials catalysts still need to be further optimized [103,104]. When introducing the N element, introducing B, S, P, F and other elements into double- or even multi-element doping can further enhance ORR activity, mainly because multiple doping groups provide more active sites, and an appropriate doping ratio can enhance the synergistic effect [105]. Choi. prepared B, P and N co-doped bamboo carbon nanotubes (BPNDC) [106]. The bamboo configuration is conducive to exposing more graphitized edges and increasing the active site of ORR, which can effectively improve the ORR performance.

The multi-doped carbon materials have good durability and resistance to methanol poisoning. The catalytic activity of ORR in alkaline media is close to that of commercial Pt/C catalysts, but the activity in acidic media needs to be improved [7]. In addition, the catalytic mechanism of ORR in multi-doped carbon materials is still unclear.

Table 1 demonstrates recent progress in the development of advanced carbon-based metal-free ORR electrocatalysts through the co-engineering of various defects and heteroatom dopants. In carbon materials, the strategy of single heteroatom doping has been proven to enhance catalytic performance. In addition, co-doping with multiple heteroatoms (such as nitrogen–sulfur, nitrogen–phosphorus, nitrogen–boron, etc.) can further improve the catalytic performance of carbon materials due to the synergy effect produced by electron interactions between different dopants. Interestingly, these electrocatalysts can exhibit ORR performance that is comparable, and in some cases even superior, to that of state-of-the-art Pt/C catalysts. This further confirms the importance of the synergistic promotion effect between defects and dopants. Although carbon-based nanomaterials can serve as catalysts on their own, there still exists a certain gap in catalytic efficiency between non-metal and metal catalysts. Many researchers in this field attempt to enhance the catalytic activity of carbon catalysts through heteroatom doping or functional modification. However, as the inherent defects in carbon materials increase, the stability of the catalyst decreases, leading to lower recycling rates.

## 5. Summary and Prospect

Nonmetallic catalysts have a low cost, good stability and resistance in oxygen reduction reactions. To provide references for the design and synthesis of non-metallic catalysts, this paper systematically reviews the research progress of non-metallic catalysts in oxygen reduction reactions and focuses on their synthesis and characterization. Although the related research has made great progress, there are two problems to be solved: (1) the catalytic activity of ORR in an acidic medium is relatively low, which cannot meet the requirements of fuel cells; (2) the catalytic mechanism of ORR in carbon materials doped with different atoms is still unclear. On the whole, except for nitrogen doping, other heteroatomic-doping contents are very low, which greatly affects the performance of related materials, so it is necessary to develop new preparation methods and explore new methods, which will lead to the faster commercialization of fuel cell catalysts.

At present, such catalysts have high catalytic activity under alkaline conditions but not under acidic conditions. The main reasons can be attributed to the following three points: (1) an acidic medium is more corrosive than an alkaline medium; (2) nitrogen-containing active sites are easily protonated, leading to failure in the process of catalytic ORR; (3) heterogeneous element doping is mostly realized through heat treatment, but it is easy to decompose at high temperatures, resulting in a low doping amount and limiting the improvement of catalytic activity. How to improve the performance of heteroatomic-doped carbon nanotubes catalysts under acidic conditions is still a great challenge.

## Figures and Tables

**Table 1:** Typical defect- and heteroatom doping-co-engineered non-metal nanocarbon electrocatalysts and their key ORR parameters in an alkaline medium.

Catalyst	Loading (mg cm^−2^)	E_0_ (V vs. RHE)	E_1/2_ (V vs. RHE)	References
Pyridinic N-doped hydrogen-substituted graphdiyn	0.4	1.02	0.85	[10]
S-doped carbon nitride	0.5	0.93	0.83	[24]
N-doped carbon nanoribbons	12.39	0.99	0.87	[42]
N-doped carbon coaxial nanocables	0.255	0.99	/	[48]
N-doped amorphous carbon	1.00	0.934	0.78	[53]
N-doped graphene oxide	0.6	1.1	0.84	[56]
N-doped graphene hollow microspheres	0.337	0.934	0.777	[57]
B-doped graphene	0.040	0.915	/	[71]
3D S-doped graphene	0.2	1.01	/	[87]
P-doped ordered mesoporous carbons	0.79	0.86	/	[74]
N, S-co-doped porous exfoliated carbon nanosheets	0.181	0.97	0.83	[89]
N, S-co-doped graphene	/	0.91	/	[90]
N, P-co-functionalized three-dimensional porous carbon networks	0.2	0.92	0.78	[100]
B, N- co-doped graphite layers	0.283	1.01	0.82	[96]

## Data Availability

All relevant data are within the paper.
